# Recruitment and Retention Strategies Among Racial and Ethnic Minorities in Web-Based Intervention Trials: Retrospective Qualitative Analysis

**DOI:** 10.2196/23959

**Published:** 2021-07-12

**Authors:** DaSol Amy Hwang, Alex Lee, Jae Min Song, Hae-Ra Han

**Affiliations:** 1 Johns Hopkins University School of Nursing Baltimore, MD United States

**Keywords:** recruitment and retention, web-based intervention, clinical trial, Korean American, racial/ethnic minority

## Abstract

**Background:**

Racial and ethnic minority groups are underrepresented in health research, contributing to persistent health disparities in the United States. Identifying effective recruitment and retention strategies among minority groups and their subpopulations is an important research agenda. Web-based intervention approaches are becoming increasingly popular with the ubiquitous use of the internet. However, it is not completely clear which recruitment and retention strategies have been successful in web-based intervention trials targeting racial and ethnic minorities.

**Objective:**

This study aims to describe lessons learned in recruiting and retaining one of the understudied ethnic minority women—Korean Americans—enrolled in a web-based intervention trial and to compare our findings with the strategies reported in relevant published web-based intervention trials.

**Methods:**

Multiple sources of data were used to address the objectives of this study, including the study team’s meeting minutes, participant tracking and contact logs, survey reports, and postintervention interviews. In addition, an electronic search involving 2 databases (PubMed and CINAHL) was performed to identify published studies using web-based interventions. Qualitative analysis was then performed to identify common themes addressing recruitment and retention strategies across the trials using web-based intervention modalities.

**Results:**

A total of 9 categories of recruitment and retention strategies emerged: authentic care; accommodation of time, place, and transportation; financial incentives; diversity among the study team; multiple, yet standardized modes of communication; mobilizing existing community relationships with efforts to build trust; prioritizing features of web-based intervention; combined use of web-based and direct recruitment; and self-directed web-based intervention with human support. Although all the studies included in the analysis combined multiple strategies, prioritizing features of web-based intervention or use of human support were particularly relevant for promoting recruitment and retention of racial and ethnic minorities in web-based intervention trials.

**Conclusions:**

The growing prevalence of internet use among racial and ethnic minority populations represents an excellent opportunity to design and deliver intervention programs via the internet. Future research should explore and compare successful recruitment and retention methods among race and ethnic groups for web-based interventions.

**Trial Registration:**

ClinicalTrials.gov NCT03726619; https://clinicaltrials.gov/ct2/show/NCT03726619.

## Introduction

### Background

Health research creates generalizable scientific knowledge, leads to positive changes in health policy, and improves human health and health care [1,2]. However, these benefits are not shared equally, particularly among the racial and ethnic minority groups in the United States, as they are vastly underrepresented in biomedical research. For example, racial and ethnic minority participation in cancer clinical trials was approximately 20% compared with 80% of White counterparts in 2018 [3], and this has yielded only a 2% increase in the past two decades. Racial and ethnic minorities constituted 40% of the US population in 2018 [4], and this number is projected to continue to rise, making up more than 50% of the total population by 2050 [5]. The National Institutes of Health policy states that all National Institutes of Health–funded clinical research must include and address minority groups and their subpopulations in their research objectives, design, and recruitment [2]. Therefore, effective recruitment and retention strategies for racial and ethnic minority populations must be identified, evaluated, and used to reduce the underrepresentation of racial and ethnic minorities in health research.

A systematic review of studies addressing research participation among racial and ethnic minorities [6] revealed various factors salient to racial and ethnic minority participation in research. The most common barriers across all 4 ethnic minority groups—Blacks or African Americans, Asian Americans, Latinos, and Pacific Islander—were mistrust (77%), followed by time and financial constraints (45%), fear of unintended outcomes (31%), limited access to information (31%), stigma about participating in research (27%), fear of discrimination related to health insurance coverage (7%), and fear of deportation and concern about their immigration status being affected (5%). In addition, a substantial number of racial and ethnic minority groups experience logistical problems such as transportation, time commitment, and financial means to make office visits [7], lack of social support, low socioeconomic status, lack of culturally targeted or sensitive information, and education about the study [8]. Finally, provider biases against minority patients, such as assumptions about patient preferences, knowledge, and compliance, especially when accompanied with language barriers, may contribute to a provider’s failure to offer opportunities for their patients to participate in research [7]. This lack of research participation could hinder racial and ethnic minority individuals from exploring different treatment options and receiving high-quality care while contributing to future generations.

With the rapid advancement of technology and its convenience, access to the internet is highly prevalent. According to the Health Information National Trends Survey by National Cancer Institute in 2007 [9], 70% of 4092 Health Information National Trends Survey respondents indicated that they had access to the internet, excluding work and public spaces, and there was consistent use of social networking sites among racial and ethnic minorities. According to the World Data sourced by the World Bank, the share of the United States population using the internet increased from 69% in 2006 to 76% in 2016 [10]. Similarly, a recent report from the Pew Research Center showed that the internet use penetration rate is equally, if not more rapidly, increasing among Blacks and Hispanics by 17% and 15% between 2010 and 2019, respectively, as compared with that of their White counterparts, which increased by 14% [11]. The growing prevalence of internet use, particularly among racial and ethnic minority populations, represents an excellent opportunity for researchers to design and deliver intervention programs using technology. Indeed, an increasing number of clinical trials have used the internet as the main mode of intervention delivery [12]. However, there is a lack of research addressing recruitment and retention strategies among racial and ethnic minority populations in web-based intervention trials, suggesting an important gap in the literature.

### Objective

e-CHECC-uP (e-Community–Based Health Literacy–Focused Intervention for Cancer Control) is a recently completed randomized controlled pilot trial (trial registration: ClinicalTrials.gov NCT03726619) in which a web-based intervention was used to promote breast and cervical cancer screening among Korean American women. The purpose of this paper is to describe our experiences and lessons learned in recruiting and retaining Korean American women for the e-CHECC-uP study. We also compare our findings with the strategies reported in relevant published studies to enhance our understanding of the recruitment and retention of ethnic and racial minorities in web-based intervention trials.

## Methods

### Description of the e-CHECC-uP Study

e-CHECC-uP was a community-based randomized controlled pilot trial designed to promote mammogram and Papanicolaou test screening practices among Korean American women. Details regarding the study design and intervention are described elsewhere. Briefly, the e-CHECC-uP intervention consisted of web-based health literacy education followed by phone counseling for 6 months. The study inclusion criteria were as follows: (1) Korean American women aged between 21 and 65 years, (2) residing in the Baltimore-Washington metropolitan area, and (3) who were overdue for an age-appropriate mammogram (ie, had not had a mammogram within the past year for women aged 40-54 years or past 2 years for women aged 45-65 years) [13] or a Papanicolaou test (ie, had not had a Papanicolaou test within the last 3 years) [14]. Our recruitment goal was 40 Korean American women (20 in the intervention arm and 20 in the control arm) for this pilot study.

Upon approval of study procedures by the Johns Hopkins Medicine Institutional Review Board, the study team worked with ethnic churches in the target area to identify potential participants by posting flyers, working directly with pastors to make an announcement about the study, and by word-of-mouth among the potential participants. Specifically, after the trained bilingual research assistants identified and contacted the spokesperson (pastor, office secretary, or community health worker) from each church via phone calls and emails, a study coordinator sent an introductory email with a study flyer in Korean and English and a summary of the study for the church bulletin board announcement. Once approved by the church leadership board, which often included pastors and older adults, in-person baseline visits were scheduled at their convenience, usually during lunchtime or after church service on Sundays. Trained bilingual research assistants met potential participants at their church to explain the study procedure, obtain informed consent, and provide study-related assistance. The enrolled participants completed the study surveys at baseline and at 3 months and 6 months. To address scheduling challenges and time constraints for some potential participants, we also offered an option to complete the consent process and data collection on the web. Every participant received financial incentives at each data collection (US $20 each at baseline and at 3 months and US $40 at 6 months). At the conclusion of the study survey at 6 months, women in the intervention arm were invited to participate in postintervention phone interviews. Each interview lasted for 15-30 minutes. Those who participated in the postintervention interview received additional US $30.

### Sources of Data

We used multiple data sources for the e-CHECC-uP trial to address the study purpose, including the study team’s weekly meeting minutes, transcripts for postintervention interviews, recruitment and retention tracking logs, survey reports, and other study forms recording contacts with participants such as phone counseling logs. In addition, key lessons learned for recruitment and retention from published studies using web-based interventions were identified and compared with the e-CHECC-uP strategies. Our search was conducted in April 2020, and the strategy involved 2 electronic databases: PubMed and CINAHL. In consultation with a health science librarian, we used the following search terms: *research subject recruitment* OR *research subject retention* OR *recruitment strategies* OR *retention strategies* OR *research participation* AND *web-based* OR *Internet* OR *computers* AND *ethnic groups* OR *ethnic minority*. We included the articles that involved interventions with technology or the internet for racial and ethnic minority populations in the United States, discussed recruitment and retention strategies, and were published in the last 10 years in English. We excluded the studies in which involvement of technology was limited to the recruitment method only. Our search yielded 163 articles, and following the abstract and full-text screening, 7 articles, including 2 systematic reviews, were identified as being relevant to our discussion.

### Analysis

Using diverse sources of data, recruitment and retention strategies used in the e-CHECC-uP trial were described, discussed, and solidified among the research team members. Descriptive statistics such as means, frequencies, and percentages were used to summarize recruitment and retention rates in the e-CHECC-uP trial. A thematic analysis of the intervention participant interview was conducted to identify the common experiences of Korean American women with the study intervention in the trial.

## Results

### Characteristics of e-CHECC-uP and Other Relevant Studies

Table 1 summarizes the key characteristics of the e-CHECC-uP study and the 7 relevant studies that used web-based intervention, as identified from our electronic search. The e-CHECC-uP study participants were recruited over a span of 6 months (July to December 2019) from 5 Korean churches in the Greater Baltimore area, and 72% (52/72) women who were screened for this study were considered as eligible for enrollment. However, 8 of those eligible women refused to enroll in our study because of time constraints (n=4), health reasons (n=1), lack of interest (n=1), and reluctance to undergo a cancer screening test in the United States because of inconvenience (n=1). In addition, we could not reach 4 eligible women after initial contact, yielding 40 women enrolled in the study (77% response rate). The majority of women (n=38) were enrolled face-to-face and 2 were enrolled on the web. In total, 34 participants (intervention: n=15 and control: n=19) completed the final study assessment at 6 months (retention rate 85%). Reasons for withdrawal included time constraints (n=3), participation burden (n=1), change of mind (n=1), and lost contact (n=1). The scope of web-based interventions included in the relevant studies addressed health promotion and participant education. The follow-up period for these studies ranged from 3 weeks to 24 months. Of the 7 relevant studies, 4 randomized controlled trials (RCTs) and 1 RCT-based secondary analysis focused on various racial and ethnic minority groups, including Asian Americans (Chinese, Korean, and Japanese, specifically), African Americans, and Black or Latino. The interventions included the Fitbit device, weekly interactive web-based comics with text and email reminders, web-based education involving in-person visits, and social media websites using a mobile chat function in participants’ mother language. There were 2 systematic reviews of e-mental health interventions among Asian American women and mobile health (mHealth) research participation among African Americans, including 79 studies and 56 studies, respectively.

**Table 1:** Characteristics of studies with web-based interventions involving racial and ethnic minorities.

Reference	Study design; goal	Sample	Follow-up period	Intervention	Recruitment and retention methods with rates
Hwang et al (unpublished, 2021) e-CHECC-uP^a^	RCT^b^ (2 arms); to promote mammogram and Papanicolaou test screening among Korean American women	Korean American women who were overdue for an age-appropriate mammogram and/or Papanicolaou test screening (n=40)	6 months	Intervention: web-based health literacy–focused education followed by monthly phone counseling+cancer screening brochure; control: cancer screening brochure	Posting flyers or working directly with pastors to make study announcements in ethnic churches and word-of-mouth; 77% recruitment yield rate; the retention rate was 85% at 6 months
Chee et al (2019) [15]	RCT (3 arms); to promote physical activity among Asian American women	Chinese and Korean women who were aged 20-60 years, who were fluent in English or native language, and who had internet access (n=165)	3 months	Group 1: web-based physical activity promotion; group 2: 1+Fitbit Charge; group 3: 2+Fitbit+office visits	Contacted Asian American groups and communities to announce about the study via email or website; 76% recruitment yield rate. Retention rate was 23% at 3 months (22% for group 1, 37% for group 2, and 0% for group 3)
DeFrank et al (2019) [16]	RCT (2 arms); to decrease obesity risk among urban minority youth	Black or Latino child (aged 9-12 years)-parent dyads, with child BMI percentile of 5% or higher and internet access (n=89)	3 months	Intervention: a 6-chapter interactive web-based nutrition comic (1 chapter per week) combined with weekly text or email messages and reminders from comic characters and self-assessments of how they did on their weekly health goals; control: 6 web-based newsletters with nutrition-related content (1 per week) with weekly text or email messages and self-assessments	Recruitment rates yielded by the various recruitment methods: community events (39%), community tabling or handing out flyers (34%), friends or word-of-mouth (15%), and community clinic referrals (12%); the retention rate was 84% at 3 months
Staffileno et al (2017) [17]	RCT (2 arms); to evaluate the feasibility of web advertising as a recruiting modality for a healthy lifestyle behavior change intervention targeting young African American women	African American women (aged 18-45 years) with untreated prehypertension and internet access (n=35)	12 weeks	Web-based education (12 modules) focusing on either physical activity or Dietary Approaches to Stop Hypertension	Study flyers, tabletop cards, blood pressure screenings at health fairs, and clinic referrals. Web-related methods, such as Facebook, craigslist, university website, intranet, and “on-hold” telephone line, were also used; for 18 months, 176 inquiries received, and 35 women met the criteria and enrolled (20% overall rate) with rates by different recruitment methods used: employee-in-services (100%), university website (44%), flyers or tabletop cards (32%), word-of-mouth or physician referral (25%), blood pressure screenings, (15%), Facebook or Craigslist (13%), and clinics (12%)
Im et al (2020) [18]	RCT (2 arms); to determine the effect of a technology-based education and coaching intervention on improving Asian American breast cancer survivor experience	Asian American breast cancer survivors (n=94)	3 months	Intervention: 3 social media websites with preferred language chat function about breast cancer; web-based education sessions that were culturally tailored (ie, acupuncture for Chinese and red ginseng for Koreans); and web-based cultural resources and the American Cancer Society website for 3 months; control: use of the American Cancer Society website for 3 months	Social media groups, internet communities or groups, and communities or groups of Asian Americans using communication apps that are well known to the subethnic groups (weChat for Chinese, KakaoTalk for Korean, and Line for Japanese); recruitment and retention rates not reported
Reyes et al (2018) [19]	Systematic review; to address challenges, barriers, and strategies of conducting e-mental health intervention research among Asian American women	79 studies related to mental health services (not all specific to Asian American women)	No follow-up period discussion provided	No discussion on the scope and characteristics of the interventions included in the review	Participants in the studies included in the review recruited from clinical settings were less likely to complete the program than those recruited from nonclinical settings. Otherwise, no other specific information on recruitment and retention was provided; recruitment and retention rates not discussed
Im et al (2016) [20]	Secondary analysis of an internet-based intervention study using RCT with Asian American breast cancer survivors^c^; to identify practical issues in web-based recruitment of Asian American breast cancer survivors	Asian American (Chinese, Korean, and Japanese) breast cancer survivors (n=57)	3 months	Intervention: used both the internet cancer support group and the internet resources (those related to daily life and those by the American Cancer Society); control: used only the internet resources related to daily life (eg, news in Asian countries and Asian businesses in the United States) and the American Cancer Society website	Study announcement via internet breast cancer support groups, Facebook, and WeChat (reached 8283 potential participants but yielded only 69 study website clicks); 171 internet breast cancer support groups identified, and 6 of them responded to study team email request to post study announcement, with 0.2% response rate of potential participants; retention rates were approximately 50% across the 3 subethnic groups
James et al (2016) [21]	Systematic review; to review participation of African Americans in eHealth or mobile health interventions	Studies that were published in English, conducted in the United States, included African American adults (aged ≥18 years), and specified the type of technology used (n=56)	Ranges from 3 weeks to 24 months	No description of the scope and characteristics of eHealth or mobile health interventions included in the review	Recruitment settings or methods used in the studies included in the review: clinics or health care facilities, 52% (29/56, of studies included); flyer and newspaper, 25% (14/56); websites, listserv, email, or postal mailings, 15% (8/56); churches, n=2; college campus, n=3; no recruitment and retention rates reported

^a^e-CHECC-uP: e-Community–Based Health Literacy–Focused Intervention for Cancer Control.

^b^RCT: randomized controlled trial.

^c^Original study citation not provided.

### Recruitment and Retention of Racial and Ethnic Minorities in Web-Based Intervention Trials

#### Recruitment and Retention in Other Web-Based Intervention Trials

Analysis of various data sources revealed several key strategies and lessons learned relevant to the recruitment and retention of racial and ethnic minorities in web-based intervention trials (Textbox 1). These included authentic care; accommodation of time, place, and transportation; financial incentives; diversity among study team; multiple, yet standardized, modes of communication; mobilizing existing community relationships with efforts to build trust; prioritizing features of web-based intervention; combined use of web-based and direct recruitment; and self-directed web-based intervention with human support.

**Key strategies for the recruitment and retention of racial and ethnic minorities from studies using web-based interventions.**

**Authentic Care**

- Active engagement individualized for participants (eg, assigning study staff to participants and ongoing study process quality assessment) [16,19,21]
- Accommodating participants’ family members (eg, separate hospitality room, entertainment, and snacks) [16]
- Continuous showing of genuine care with respect (e-Community–Based Health Literacy–Focused Intervention for Cancer Control [e-CHECC-uP] study)

**Accommodation of Time, Place, and Transportation**

- Flexible scheduling (eg, 7 days a week, including after school and evenings, weekends) [16], (e-CHECC-uP study)
- Accessible location and providing transportation for in-person data collection in consideration of low-income and minority population [16,21] (e-CHECC-uP study)
- Changing from in-person visits to fully remote work to minimize in-person contact while adapting to recommended health care guidelines during COVID-19 for participants’ health and safety (e-CHECC-uP study)

**Financial Incentives**

- Incentives matching with study participants’ interest [16]
- US $50 electronic gift cards [18]
- US $20 gift card at each in-person visit [17]
- Incentives periodically or at the end [21] (e-CHECC-uP study)

**Diversity Among Study Team**

- Multilingual or bilingual research staff reaching out and delivering the intervention to a population with low socioeconomic status and low English proficiency [16,18,20] (e-CHECC-uP study)
- Study team members with technological proficiency to enhance the participant’s learning and use of web-based content (e-CHECC-uP study)

**Multiple, Yet Standardized, Modes of Communication**

- Multiple modes of communication (eg, phone, email, and text messaging) to provide a diverse platform for efficient interactions between the study team and study participants [16,19] (e-CHECC-uP study)
- Standardized and unified communication system (eg, dedicated study phone line or email address) [16,19]
- Visit reminder protocol via automated text messaging (e-CHECC-uP study)

**Mobilizing Existing Community Relationships With Efforts to Build Trust**

- Working with community consultants and leaders (eg, listening to a pastor’s advice to visit the community site) [15] (e-CHECC-uP study)
- Using a peer advocate group to enhance retention among patients with breast cancer [21]
- Community-based recruitment (eg, targeted flyers posted at local businesses, partnerships with community- or faith-based organizations, and event tabling) [16] (e-CHECC-uP study)
- Dissemination of study progress with sharing of research experiences and findings through writing or voice recording [22] or video recording (e-CHECC-uP study)

**Prioritizing Features of the Web-Based Intervention**

- Prioritizing features of the web-based intervention (ie, ease and convenience of use, perceived helpfulness, and likelihood of future use) [19]
- Developing thorough guidelines regarding interventions with technology in participants’ own languages with precreated IDs and passwords [15] (e-CHECC-uP study)

**Combined Use of Web-Based and Direct Recruitment**

- Web-based strategies (eg, university website or Craigslist) have greater potential to reach a larger number of people with less financial and human resources but are not as effective as traditional in-person strategies [17]
- A combination of internet setting and physical community settings to recruit target sample size [20] (e-CHECC-uP study)

**Self-Directed Web-Based Intervention With Human Support**

- Having participants monitor their own progress with goals of building confidence and persistence and solving issues and concerns [18]
- Human support (eg, professionals, peers, and community health workers) to promote participant engagement in the intervention activities [19] (e-CHECC-uP study)

#### Authentic Care

The Belmont Report issued in 1978 articulates 3 ethical principles that researchers should abide by: respect for persons, beneficence, and justice. Applications in the research setting include informed consent, assessing the participants’ comprehension and voluntariness, providing additional protection for those with diminished autonomy, maximizing benefits and minimizing harm to study participants, and fair distribution of research benefits and burdens among diverse populations [23]. As such, authentic care can be translated into the active engagement of study participants in the study process. According to Woolf et al [24], authentic engagement not only enhances the study participants’ understanding of data but also ensures their participation as stakeholders and active community agents to further disseminate study findings, develop supplemental resources, and collaborate with local leaders and policy makers. The collective impact of authentic care delivery is rooted in beneficence. Although authentic care may not be measurable by numerical data, our participants in the e-CHECC-uP trial stated their appreciation during the postintervention interview. A participant shared:

...I felt like I was being taken care of...I saw how you were trying hard to encourage me and share with me what you know.

Another participant said:

Honestly, going to the hospital takes a toll...I really did not want to go so I kept delaying my appointment, but I felt good after the [cancer screening] visit. Then I thought ‘wow, this push was all for me, not for [the research team]...

The shared experience of our participants shows that authentic care in our communication and interactions was a vital part of their participation as a source of empowerment of their own preventive health care management and knowledge. In an RCT to decrease obesity risk among Black and Latino children (aged 9-12 years), DeFrank et al [16] reported assigning dedicated research staff as one of the key strategies. By doing so, DeFrank et al [16] noted that 81% of children at 3 months postintervention were very satisfied or extremely satisfied with how the study staff communicated and interacted with them.

#### Accommodation of Time, Place, and Transportation

Korean Americans who are first-generation immigrants experience busy immigration lifestyles, involving several family obligations, financial responsibilities, and social status, because of which health care management is often neglected [25]. Several church members refused to participate in the e-CHECC-uP trial because of their parental and familial duties. When proposed with alternative midweek and weekend options, some eligible women stated that it was simply too inconvenient to schedule meetings because of their children’s schedules. Likewise, 4 out of 6 participants who withdrew from the study shared that their main reason was time constraints or that the study was *too burdensome* to continue. A participant stated that “it was a bit overwhelming...to make time for the phone counseling since I have other work to do,” which further shows the participant’s lack of time because of a busy lifestyle, not because of their lack of interest in the study itself. To reach and better retain participants, our research team accommodated controllable measures such as study visits, call times, and meeting places according to the individual preferences of the study participants. For example, most study visits for both recruitment and follow-up data collection were made on Sundays at a church as a preferred and most convenient community location for the participants. DeFrank et al [16] also noted that offering flexible hours was one of the factors associated with a high retention rate of 84%. Flexible scheduling was further enhanced by a web-based survey (Qualtrics) for data collection. We decided to use Qualtrics to allow willing participants to complete their follow-up study surveys remotely, at their own time and convenience. Qualtrics proved to be a viable resource, especially with the onset of COVID-19. In March 2020, we halted all in-person research visits to prioritize safety, given the increased risk of exposure to COVID-19. Despite having the added responsibility of uploading voice recordings for the reading test section for health literacy assessments, participants in the e-CHECC-uP trial were compliant in collaborating via Qualtrics. Using Qualtrics, we were able to retain all remaining participants at the onset of COVID-19 and achieved a retention rate of 85% (34/40).

#### Financial Incentives

We used an electronic gift card to partially compensate participants for their time spent on the study activities. A participant noted her experience:

If I participate, I get something in return. It is a total motivation.

Another participant shared that it was a good side income to buy lunch for her children. In a systematic review of 56 studies focusing on African American participation in eHealth or mHealth intervention studies, James et al [21] found that 25 of the included studies offered some type of incentive, ranging from monetary (US $10-US $175) to nonmonetary incentives such as prepaid cell phones, free hypertension medication, and gym membership. Although the studies that provided incentives yielded a higher retention rate, James et al [21] recommended that researchers carefully determine the type and structure of incentives tailored to specific populations, communities, and targeted health behaviors. DeFrank et al [16] also noted that incremental monetary compensation through gift cards, which was selected based on formative research by surveying participants and the community, was an effective recruitment and retention strategy.

#### Diversity Among the Study Team

Language barriers remain one of the prevailing challenges among immigrants and are often associated with socioeconomic disadvantage, higher poverty, and uninsured rates. In 2012, 34% of the immigrant population in the United States was limited English proficient compared with 9% of the total population. According to the Association of Asian Pacific Community Health Organizations [26], Korean Americans reported the second highest (46%) limited English proficient rates, followed by Vietnamese (53%), and had the highest rate of reporting poor patient-physician communication (34%) compared with other subethnic groups. Our findings were similar, with 50% (20/40) of our participants reporting that they were unable to make phone calls in English and 58% (23/40) reporting that they would not be able to go to an American hospital without an interpreter. Our study team members were diverse in terms of Korean fluency, technological competency, and gender. In particular, the bilingual study staff were instrumental in developing partnerships with study sites and community resources, disseminating critical information such as details on the consent forms during recruitment, and providing phone counseling for participants. A technology-based study on the practical issues in recruitment and retention of racial and ethnic minorities reported that many of their study participants were only comfortable participating in the study because they could use their heritage languages and were coached or supported in their heritage languages throughout the intervention [18]. In addition, the technologically inclined staff on the e-CHECC-uP study team were vital for troubleshooting issues with the web-based education modules, developing user-friendly remote surveys with prerecorded web-based instructions, and facilitating lines of e-communication with research participants via automated text messaging. Although 90% (36/40) of our participants reported having access to the internet at baseline, 70% (28/40) of the intervention women agreed or strongly agreed that they would need the support of a technical person to be able to use the product. Finally, despite initially operating with the notion that the study population would best respond to female study team members, as the e-CHECC-uP study focuses on women's cancer screening, we discovered that there were no observable differences in participant interaction or receptivity with male study team members. An internet-based study reported that racial and ethnic minority women responded well to race-concordant research assistants [20]. Therefore, we believe that trust was built between the study participants and the study team members, as they were of the same race and ethnicity.

#### Multiple, Yet Standardized, Modes of Communication

We used multiple modes of communication to accommodate individual communication preferences. Although phone calls were initially the main method of communication, we expanded our platforms to include text messaging and email after having failed to reach some study participants. A recent systematic review [19] revealed that adding email and telephone support to existing internet-based mental health interventions led to high completion rates and favorable mental health outcomes. Overall, text messaging was the most effective and preferred method to reach Korean American women in the e-CHECC-uP trial because it is quick to read and more accessible than phone calls or email. In fact, 39 out of 40 participants (97.5%) at baseline reported that they text at least once a week, and 34 participants (85%) reported that they text daily. Consequently, we scheduled general updates and reminders to be sent via text messaging using Qualtrics. We set up a study visit reminder protocol that involved an automated text message related to our scheduled follow-up data collection visit on Sunday that was sent out to participants a week before; participants would respond Y (yes) or N (no) to the text message to indicate their availability. If they responded Y, the study team would send a reminder text message on Friday and another on Sunday morning. Only when participants responded N were they contacted via phone to schedule a separate visit or to complete the study survey on the web. We received a 38% response rate using Qualtrics for 3-month and 6-month reminder text messages. DeFrank et al [16] also reported that a well-designed, standardized communication system was a key recruitment and retention strategy. The study staff used a single phone number for all calling and texting, a simplified study email address accessible to all study staff, and a single calendar as a central scheduling platform. All avenues of communication with the research participants were monitored and evaluated every week to develop improvement strategies as challenges surfaced. These interventions contributed to high survey completion rates throughout the study (84% at 3-months postintervention), high satisfaction rates (81%), and positive study experiences (97% of the participants felt that they received enough study information, and 80% (32/40) of the participants felt that their questions were answered properly).

#### Mobilizing Existing Community Relationships With Efforts to Build Trust

Before active recruitment in July 2019, the study team retrieved a list of ethnic churches in the Baltimore-Washington metropolitan area from the principal investigator’s previous studies with the Korean American population. Additional churches from Google searches and word-of-mouth were identified. The word-of-mouth strategy is a well-known method for participant recruitment [27-30]. In addition, we mobilized a community health worker who had worked with the principal investigator in previous research studies. A community health worker is a “frontline public health worker who is a trusted member of and/or has an unusually close understanding of the community served” [31]. The involvement of community consultants and leaders in research not only increased positive recruitment results but also provided vital advice on how to approach participants and gain their trust [15]. Our community health worker helped us to build rapport with the pastor and church members, provided a brief introduction about the study, gathered those who showed interest, and advised on the timing of visits and how to approach potential participants. In addition, the active involvement of community leaders was impactful in recruiting and retaining participants for the e-CHECC-uP trial. For example, some pastors made verbal announcements at the end of the church service going above and beyond just posting our study information on their church bulletin board. Our study team members reflected and agreed that the participants from those churches were more engaging, asking questions, and being responsive to our contact. Our study team made additional efforts to build a collaborative relationship with the church. We were in consistent communication with the pastors via timely updates and greeting emails. This was especially effective in the beginning phase of the COVID-19 pandemic, as we periodically checked in with our study participants and sent emails about our study progress and next steps in the face of uncertainty and restricted study site visits. To this end, we plan to disseminate our study findings to the churches, as we highlighted in our study completion letter to the participants. Sharing study results with community participants may further reinforce ethics and communication between the research and the community, reducing the stigma against clinical trials and population [32].

#### Prioritizing Features of Web-Based Intervention

Before the active recruitment of the e-CHECC-uP study participants, we conducted a usability test with 8 Korean American women who shared similar characteristics to the inclusion criteria used in the pilot clinical trial. For 5 months, we met with the women biweekly to hold their feedback to each developmental stage of the e-CHECC-uP educational website with the website developer. The features of web-based intervention programs were assessed to identify the ease and convenience of use, perceived helpfulness, and likelihood of future use. According to a recent systematic review, prioritizing the web-based intervention development enhanced participants’ engagement [19]. The amount of time that participants committed to complete web-based activities was associated with the perceived study burden. In a study, a short average length of time lowered the participant burden and resulted in a high satisfaction rate [16]. Moreover, we provided a 1-sheet user guide to the participants in the intervention arm. This document was also available in Korean and English. As it summarizes the intervention module and highlights important features to increase participants’ understanding and knowledge on the research topic, developing thorough written guidelines regarding interventions with technology is essential [15].

#### Combined Use of Web-Based and Direct Recruitment

Challenges with direct recruitment and enrollment, such as time constraints, motivated us to expand our recruitment approaches using a web-based recruitment strategy. Our web-based recruitment efforts yielded 100% success when they were used with interpersonal engagement. Interpersonal engagement in a community-based approach at the recruitment stage is vital for high enrollment and retention rates [21]. We contacted our web-based participants through previously established lines of in-person contact, engaged and communicated with them via texts and emails, and enrolled them through Qualtrics. This multilevel approach of using both web-based and direct recruitment allowed us to meet our target sample size and resulted in a more diverse participant pool, as this strategy was successful for relatively younger, bilingual participants who were in different life stages than the majority of our participants. Similarly, a systematic review [21] of mHealth intervention studies with African Americans suggested that a multilevel recruitment approach that considers culture, learning styles, and personal engagement may result in a larger, more diverse participant pool than using a single recruitment strategy.

#### Self-Directed Web-Based Intervention With Human Support

As more internet-based education and materials are offered, the importance of human support can be overlooked. However, human support and privacy or anonymity are still valued when delivering web-based interventions [19]. The e-CHECC-uP study included exclusive web-based intervention, including websites with videos on expected conversations and background information regarding mammograms and cervical cancer screening. Participants still found our study team members' assistance and monthly phone counseling to be helpful and vital to successful screening; 12 of 14 participants from the intervention group who completed the 6-month survey claimed that human support from the study team, phone counseling specifically, helped them to complete the screenings. A participant stated that the phone counseling reminded her of the importance of cancer screenings, “these studies are for my own benefits,” and she preferred talking with the study team rather than a trip to the obstetrician-gynecologist to have “deep conversation,” as her “secrets are being kept.” Adding bilingual study team members with similar cultural backgrounds increases recruitment rates and has easier access to building trust and rapport with the participants; it also promotes acknowledging the importance of web-based interventions, as the study team can include culturally inclusive examples for better understanding [18]. Providing human support via phone counseling to remind the participants about web-based resources assisted in keeping participants in the loop but still maintaining social distancing to protect them.

## Discussion

### Principal Findings

We successfully recruited and retained Korean American women in a community-based cancer prevention pilot trial with a web-based intervention. The study team used diverse strategies to promote Korean American women’s participation in the e-CHECC-uP trial. Our study’s success was largely dependent on the collaboration between the study staff and the research participants. On the basis of our analysis of the e-CHECC-uP study and other relevant studies using web-based interventions, 9 categories of recruitment and retention strategies emerged. Although we did not measure the efficacy of each individual strategy, our thematic analysis resulted in particularly rich themes related to strategies addressing the quality of the bilingual research team.

Overall, we can attest that genuine care and accommodations are among the key strategies to engage participants and enhance their acceptance of our web-based study. Postintervention interviews supported the e-CHECC-uP study participants’ trust in the study team and our genuine care. Mistrust has been noted as a major barrier to racial and ethnic minority participation in clinical trials [6]. Other successful approaches used to build trust include social networking through community engagement, such as working with community advisory boards, community health workers, and community leaders or gatekeepers [30,33-37]. In a community-based cervical cancer prevention study for African immigrant women, Cudjoe et al [36] provided education about the research process and confidentiality to promote trust in research and research teams. These recruitment methods further extend to key retention strategies, such as developing trust-building relationships and realizing the benefits associated with an intervention among participants [33,38]. Further research is needed to investigate factors contributing to differences in perceptions of trust among community stakeholders, including whether expectations regarding long-term involvement impact trust.

Active communication with study participants resulted in a high retention rate (84%) and high participant satisfaction with the study [16]. Of the 75 children who completed the survey, 68% were very satisfied or satisfied with their experience in the study, and 81% of the children were extremely satisfied or satisfied with how the study staff communicated and interacted with them [16]. Similarly, we made deliberate efforts to constantly communicate with our participants and church leaders and to disseminate study progress and findings amid the COVID-19 pandemic, ultimately establishing trust and enduring relationships. Dissemination of study findings to bring the data back to the community is an important step in the research process, particularly for trials involving hard-to-reach communities. For example, according to a survey of community members (N=226) involved in health research with academic institutions, 73% of respondents noted disseminating research results to the community in a culturally relevant and appropriate manner as one of the most relevant success indicators of community engagement [39]. Future web-based trials involving racial and ethnic minorities should consider dissemination of study progress and findings as a means to engage study participants and other stakeholders in the research process, while promoting trust in the study team.

Web-based recruitment strategies using social media platforms such as WhatsApp and Facebook have been popular for recruiting African Americans, Hispanics, and Asians [22,36]. As COVID-19 is widespread and affects potential participants’ lives, researchers should consider web-based methods to approach and retain participants. Without COVID-19, evidence suggests that the combined use of web-based recruitment with in-person strategies such as face-to-face contact and follow-up [33,35], word-of-mouth [27-30], and community-level recruitment [30,40,41] have been the most successful in recruiting racial and ethnic minorities. In addition to bilingual capacity and cultural knowledge, the e-CHECC-up team mobilized ethnic churches as the main setting for direct recruitment. Faith-based organizations serve as epicenters for social, religious, and health promotion activities across diverse racial and ethnic minority and immigrant communities, including African American, Hispanic, and Asian Americans [33,36,42,43]. Building upon existing community strengths and resources, faith-based organizations are well-suited community partners for health research and for long-term and sustainable collaboration [42]. Future web-based intervention trials should consider faith-based organizations as a key setting for the direct recruitment of racial and ethnic minorities.

Although several categories of recruitment and retention strategies identified from our analysis were also relevant to traditional in-person intervention programs, a few were unique to web-based interventions. Specifically, we found that prioritizing features of web-based intervention or use of human support were important to promote recruitment and retention of racial and ethnic minorities for web-based intervention trials. Some effective methods to create a relevant, useful web-based intervention are to work with target users during the development process [19]. Assessment of the usability of web-based intervention programs before launching the program may also increase the likelihood of its future use [44]. As more research trials progress into web-based interventions, it is important to integrate other facilitators such as cultural congruence, financial or medical benefits, convenience, and low risk in participation when proposing recruitment and retention strategies among racial and ethnic minorities [6].

### Limitations

This study has several limitations. Our study used multiple recruitment and retention strategies. Consequently, we were unable to distinguish the most successful strategies in recruiting and retaining the target sample. For example, some of our participants reported that the financial incentives contributed to their motivation to join our study, and 1 participant stated that the cash incentives were used to buy basic necessities such as her child’s school lunch. However, we did not have established metrics to measure the significance and impact of cash incentives on recruitment and retention efforts. Nevertheless, evidence strongly supports the use of multiple strategies to maximize recruitment and retention yields among racial and ethnic minorities [27-30,33,35,40,41]. The number of relevant web-based intervention trials included in our discussion might have been reduced because of our focus on recruitment and retention strategies. Similarly, the e-CHECC-uP study included a small and convenient sample of Korean American women. We expanded our discussion of recruitment and retention strategies by including other relevant studies using web-based interventions. Finally, we included studies conducted in the United States only. Therefore, the findings from the analysis may not be generalizable to racial or minority groups in other countries.

### Conclusions

In conclusion, this analysis offers useful information regarding the recruitment and retention of racial and ethnic minority populations in web-based intervention trials. There is still a dearth of literature discussing whether and how racial and ethnic minorities have been recruited in clinical trials using technology-driven intervention approaches. This study contributes to the limited body of evidence that widely known strategies are essential and constructive in recruiting and retaining minority research participants. Future studies may include exploring successful recruitment and retention methods between race and ethnic or age and generation groups regarding their various internet penetration rates, comparing different recruitment strategies with or without using technology, and assessing their efficacy.

## Abbreviations

e-CHECC-uP: e-Community–Based Health Literacy–Focused Intervention for Cancer Control
mHealth: mobile health
RCT: randomized controlled trial
